# Non‐carcinogenic and carcinogenic health risk assessment of heavy metals in cooked beans and vegetables in Punjab, North India

**DOI:** 10.1002/fsn3.3678

**Published:** 2023-09-13

**Authors:** Vineeta Kharkwal, Monika Choudhary, Kiran Bains, Mahendra Bishnoi

**Affiliations:** ^1^ Department of Food and Nutrition, College of Community Science Punjab Agricultural University Ludhiana India; ^2^ Division of Food and Nutritional Biotechnology National Agri‐Food Biotechnology Institute Mohali India

**Keywords:** beans, health risk assessment, heavy metals, vegetables

## Abstract

Beans and vegetables are consumed with cereals in India on daily basis. The aim of the study was to assess carcinogenic and non‐carcinogenic risk of heavy metals in cooked beans and cooked vegetables consumed by adults (18–59 years) and elderly (≥60 years) subjects from two districts (Ludhiana and Bathinda) of Punjab. A total of 150 households were selected from 30 different locations covering both rural and urban areas. The mean daily consumption of beans and vegetables in Ludhiana was recorded as 35.09 and 215.93 g, respectively. The corresponding figures in Bathinda were observed as 26.85 and 230.54 g. The average amounts of arsenic, cadmium, lead, and mercury were 1.44 × 10^−5^, 8.21 × 10^−5^, 1.30 × 10^−3^, and 2.61 × 10^−7^ mg/kg for cooked vegetables in urban households of Ludhiana district, respectively. The corresponding values for rural households were 1.53 × 10^−5^, 5.58 × 10^−5^, and 2.98 × 10^−4^ mg/kg while mercury was not detected. The mean chronic daily intake (CDI) of arsenic from cooked beans was significantly (*p* ≤ .001) higher in urban adult males of Ludhiana (7.74 × 10^−9^ mg/kg/day) and Bathinda (5.31 × 10^−9^ mg/kg/day) compared to their rural counterparts. Similar trend was observed in CDI of heavy metals from vegetables. The mean CDI of cadmium from cooked vegetables in urban adult females of Ludhiana (3.76 × 10^−7^ mg/kg/day) was significantly (*p* ≤ .001) higher than their rural counterparts and both urban and rural adult females of Bathinda. The study concluded that the subjects of both districts were found safe from non‐carcinogenic and carcinogenic risk associated with heavy metals present in cooked beans and vegetables, except for urban subjects and rural adult subjects of Ludhiana district who had cancer risk due to cadmium present in cooked vegetable samples.

## Abstract 2

The subjects of both Ludhiana and Bathinda districts were found safe from non‐carcinogenic and carcinogenic risk associated with heavy metals present in cooked beans and vegetables, except for urban subjects and rural adult subjects of Ludhiana district who had cancer risk due to cadmium present in cooked vegetable samples.

## 1 INTRODUCTION

Beans and vegetables are common source of carbohydrate, protein, dietary fiber, vitamins, and minerals in diet. Both are used as a staple food along with cereals. In India, beans are most commonly consumed in boiled/steamed form as *daal*, while vegetables are consumed as *sabji* or *saag*. These cooked beans and cooked vegetables are commonly consumed with *chapatis* or cooked rice in daily diet. It provides proper nutrients to the consumers. However, to fulfill consumers demand, pesticides, fertilizers, fungicides are used to increase the productivity of beans and vegetables, which ultimately increase harmful chemicals in the food. On the other side, contaminated soil, air and water also elevate the levels of heavy metals in plants (Bhatti et al., 2020; Islam et al., 2016).

Heavy metals are toxic in nature when their level increases in the body. Metals' effects on human health have been thoroughly covered in literature. Metals can have numerous negative health effects, such as dementia, neurological disorders, damage to the central nervous system, cardiovascular diseases, increases in systolic and diastolic blood pressure, stomach upset, memory loss, constipation, nausea, vomiting, listlessness and severe trembling, abdominal pain and faintness, cancer, and renal dysfunction. Cancer is the main health issue with As, Cd, Co, Pb, Ni, and Cr (Amadi et al., 2017; Rafati‐Rahimzadeh et al., 2014; Wei et al., 2017). Similarly, the contamination of beans and vegetables with heavy metals and chemicals negatively affect human health. Usually, hazardous metals enter the body of a human through ingesting (food, water), breathing (air), and skin contact (Beetseh & Onum, 2013). Irrigating vegetables with wastewater or sewage water increases the heavy metals and has been associated with many health risks (Alghobar & Suresha, 2017; Balkhair & Aahraf, 2016; Chauhan & Chauhan, 2014; Cheshmazar et al., 2018). Even vegetables grown near mining areas are also high in heavy metals (Avkopashvili et al., 2017).

Metals can be categorized as essential or nonessential. Metals can be categorized as necessary or optional. While nonessential metals, such as Ag, Al, As, Ba, Cd, Hg, Li, Pb, Sb, and Ti, have no known physiological activities in the body, essential metals, such as Co, Cr, Cu, Fe, Mn, Mo, Ni, and Zn, are needed in small amounts for body metabolism. For example, the International Agency for Research on Cancer classified Cd and Pb as human carcinogens (IARC, 2012). Pb is a pediatric toxin that can cause children to grow poorly mentally and have low intelligent quotient. It also causes deformity and deformation in unborn fetuses (Taiwo et al., 2010). Numerous illnesses, including trachea‐bronchitis, pneumonitis, pulmonary edema, kidney damage, and mortality, have been brought on by severe Cd exposure (Taiwo, 2019). Heavy metals like arsenic, cadmium, chromium, mercury, and lead have certain prescribed limits for certain foods and food products given by various national and international agencies. As, the heavy metals exceeds the given limit, consumer's health is at risk, increasing the medical cost and decreasing productivity. Therefore, identification of heavy metals sources and health risk assessment is important to safeguard public health. Hence, the present study was conducted to assess the heavy metal content in cooked beans and cooked vegetables and health risk from these heavy metals in urban and rural populations of Punjab.

## 2 MATERIALS AND METHODS

### 2.1 Study area

**FIGURE 1:**
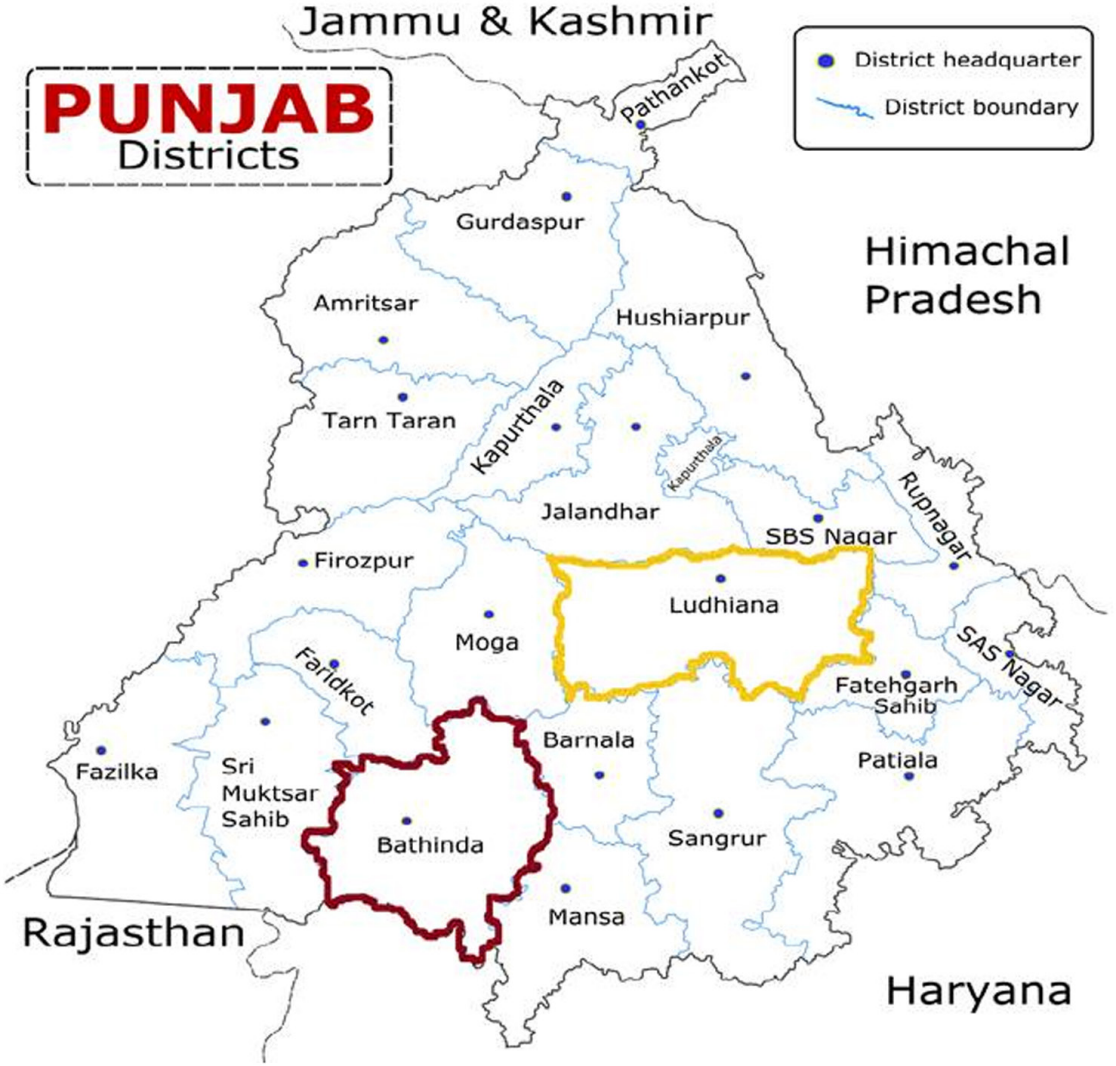
District boundary map of Punjab state.

The present study was conducted in Punjab's two major districts (Ludhiana and Bathinda) (Figure 1). A total of 13 urban and six rural locations from Ludhiana district and seven urban and four rural locations from Bathinda district were randomly selected based on the urban and rural populations of both districts. From each location, five households were randomly selected. This leads to the selection of 30 rural and 65 urban households from Ludhiana district and 20 rural and 35 urban households from Bathinda district. The subjects from the selected households were categorized based on their gender and age (adults 18–59 years and elderly ≥60 years). The general information of the subjects was obtained using the interview schedule and body weight was documented based on standard procedure (Jelliffe, 1966).

### 2.2 Food intake

The daily dietary intake of cooked beans and cooked vegetable preparations was assessed using 24‐hour recall method.

### 2.3 Collection of food samples

The cooked beans and cooked vegetable preparations were collected from each selected urban and rural household of both districts and brought to the laboratory of the department of Food and Nutrition, College of Community Science, Punjab Agricultural University, Ludhiana, Punjab, India. Beans curry (commonly known as *dal* in Indian Cuisine) is a staple food prepared for day‐to‐day lunch and dinner. There are different types of lentils or beans and each has its own cooking method. Any bean‐based recipes, it is typically pressure cooked to make it a tender and creamy texture. Later it is tempered with spices and basic vegetables like onion and tomato for a masala taste. Cooking time may vary from 10 min to 60 min. Vegetables (commonly known as *sabji* in Indian Cuisine) can be cooked in different forms such as dry, semi dry, or gravy version. This gravy is made with lightly roasted spices which give an extra flavor to recipe. The vegetable is combined with commonly available spices, spice‐powders, and other ingredients like onion, tomato, ginger, and garlic. Cooking time may vary from 10 min to 25 min. The cooked beans and cooked vegetable preparations were dried in a hot air oven at 60°C temperature till constant weight. After drying, the samples were ground using mortar and pestle and stored till further analysis.

### 2.4 Heavy metals

The heavy metal content in cooked beans and cooked vegetable preparations were analyzed at National Agri‐Food Biotechnology Institute (NABI), Mohali. In order to digest the samples in microwave digestion system (Mars6, CEM Corporation), a 50 mg powdered food sample was digested in 10 mL concentrated ICP‐MS grade nitric acid and the final volume was made to 50 mL. The digested samples were injected into the ICP‐MS system (ICP‐MS:7700×, Agilent Technologies) coupled with an auto‐sampler. The functionality of the device was ensured with frequent quality control tests and calibration checks. The validation parameters of ICP‐MS for detecting arsenic, cadmium, lead, and mercury were shown in Table 1. A standard calibration curve was prepared using varying concentrations (0, 20, 50, 100, 200, 500, and 1000 ppb) of arsenic, lead, cadmium, and mercury. Individual samples were run in triplicate and the mean value was considered for analysis. The data were analyzed using system software (Agilent Mass Hunter, Agilent Technologies Inc.).

**TABLE 1:** Method validation parameters obtained by ICP‐MS for heavy metal analysis.

Heavy metals	*R* ^2^	Calibration range (μg/kg)	Recovery (%)	LOD (μg/kg)	LOQ (μg/kg)
As	.9987	10–500	99.7	0.009	0.133
Pb	.9986	10–500	98.5	0.045	0.170
Cd	.9990	10–500	97.7	0.006	0.110
Hg	.9951	10–500	99.3	0.013	0.246

### 2.5 Health risk assessment

Health risk assessment in terms of carcinogenic and non‐carcinogenic risk of heavy metals namely arsenic, lead, and cadmium in samples of cooked beans and cooked vegetable preparations was estimated for adults and elderly subjects of urban and rural households of both districts. The non‐carcinogenic risk is evaluated using the Target Hazard Quotient (THQ). The THQ of more than 1 indicated that the subject had non‐carcinogenic risk from individual heavy metal. The following equation was used to calculate chronic daily intake (CDI) and THQ (Fathabad et al., 2018; Saleh et al., 2019):

$$\mathrm{CDI}=\left(C\times \mathrm{IR}\right)/\mathrm{BW}$$

THQ=CDI/RfD

where, CDI of a heavy metal is the daily amount of heavy metal ingested from a particular food per kg body weight (mg/kg/day), C is a heavy metal content in food (mg/kg), IR is ingestion rate (kg), BW is body weight, RfD is oral reference dose of heavy metal (mg/kg/day). The RfD for lead, arsenic, and cadmium was 0.0035, 0.0003, and 0.0001 mg/kg/day, respectively (Bhatti et al., 2020; USEPA, 2022). The RfD for mercury was not given hence THQ for mercury was not calculated.

The Hazard Index (HI) was calculated to determine the non‐carcinogenic risk from combined effect of heavy metals present in food sample (USEPA, 2015).

$$\mathrm{HI}=\sum_{i=1}^{n}\mathrm{THQ}i$$

where, THQ_i_ was the target hazard quotient of individual heavy metal present in food sample. HI of more than one indicated that individual have a higher probability of non‐carcinogenic health effects.

Carcinogenic risk was calculated to determine an individual's probability of developing cancer over a lifetime after continuous exposure (Fathabad et al., 2018; USEPA, 2005). The CR value greater than 10^−6^ indicates human carcinogenic risk (Mohammadi et al., 2019; Tepanosyan et al., 2017).

CR=CDI×CSF

where, CR is carcinogenic risk, CSF (Cancer slope factor) is the probability of one substance to increase cancer risk. CSF for arsenic, cadmium, and lead was 1.5, 6.1, and 0.0085 mg/kg/day, respectively (Bhatti et al., 2020; USEPA, 2022).

The total carcinogenic risk index (TCRI) was used to assess the carcinogenic risk from a combination of heavy metals present in food. The acceptable limit for TCRI is less than 10^−4^ (Mohammadi et al., 2019; Tepanosyan et al., 2017). It was calculated by following equation:

$$\text{TCRI}=\sum_{i=1}^{n}\mathrm{CR}i$$

### 2.6 Statistical analysis

The information obtained through the interview schedule and heavy metal content of food samples was coded and tabulated for statistical analysis using SPSS 28.0 (IBM). Descriptive statistics such as mean and standard deviation, along with percentages, were used to represent the data. Analyses of Variance (ANOVA) followed by Tukey's Honestly Significant Difference (HSD) test were used to understand the difference between groups at the level of *p* < .05.

## 3 RESULTS AND DISCUSSION

The present study was conducted in two districts of Punjab by enrolling 501 subjects from 150 households (Table 2). The body weights of adult males in both districts were higher than the reference body weight for adult males, that is, 65 kg (ICMR, 2020). Similarly, the body weights of adult females of both districts were also higher than their reference body weight (55 kg) (ICMR, 2020). The data regarding daily dietary intake of cooked beans and cooked vegetable revealed that the mean daily consumption of beans and vegetables in Ludhiana was 35.09 and 215.93 g, respectively. The corresponding figures in Bathinda were observed as 26.85 and 230.54 g.

**TABLE 2:** Number (%) of subjects included in the study to assess intake of heavy metals from Ludhiana and Bathinda districts of Punjab.

Number (%) of subjects	Ludhiana		Bathinda		Overall
	Urban	Rural	Urban	Rural	
No. of households	65	30	35	20	150
No. of locations	13	6	7	4	30
No. of subjects	206	100	120	75	501
Adult (18–59 years)					
Male	77 (44)	47 (52)	46 (49)	29 (49)	199 (48)
Female	96 (56)	43 (48)	47 (51)	30 (51)	216 (52)
Total	173 (100)	90 (100)	93 (100)	59 (100)	415 (100)
Elderly (≥60 years)					
Male	20 (61)	4 (40)	11 (41)	6 (38)	41 (48)
Female	13 (39)	6 (60)	16 (59)	10 (62)	45 (52)
Total	33 (100)	10 (100)	27 (100)	16 (100)	86 (100)
Body weight					
*Adults (18–59 years)* ^#^					
Males	73.56 ± 10.32^a^	72.49 ± 11.67^a^	79.30 ± 12.39^b^	72.38 ± 11.90^a^	0.01*
Females	64.07 ± 10.82	62.16 ± 9.18	64.94 ± 10.19	63.47 ± 10.96	0.63^NS^
*Elderly (≥60 years)* ^#^					
Males	72.5 ± 12.13	69.25 ± 15.00	72.82 ± 9.70	80.33 ± 15.38	0.49^NS^
Females	64.69 ± 11.25	65 ± 12.65	65.44 ± 14.18	59.6 ± 11.32	0.68^NS^

*Note*: Values in parenthesis are percentages. “^a–c^” means within each row with different superscripts are significantly (*p* < .05) different.

Abbreviation: NS, no significant differences.

^#^ Values are expressed as mean ± SD.

* Significant at 5% level of significance.

### 3.1 Heavy metals content of cooked beans and cooked vegetable

The heavy metal content of cooked beans and cooked vegetable preparations is presented in Table 3. The average arsenic content was 1.44 × 10^−5^ and 1.67 × 10^−6^ mg/kg for cooked beans samples collected from urban and rural households of Ludhiana district, respectively. The corresponding values for Bathinda district were 9.13 × 10^−6^ and 3.68 × 10^−6^ mg/kg. The statistical analysis revealed that arsenic content in cooked beans collected from urban households was significantly higher (*p* = .001) than the rural households of Ludhiana district. However, all of these values were within the permissible limit (1.1 ppm) specified under FSSAI (2011). In seasonal vegetable preparations, average arsenic content was 1.44 × 10^−5^ versus 1.53 × 10^−5^ and 5.73 × 10^−6^ vs. 3.95 × 10^−6^ mg/kg, for urban versus rural households of both Ludhiana and Bathinda districts, respectively. It was observed that these values were within the permissible limit (1.1 ppm) specified under FSSAI (2011). The findings of the present study corroborate with earlier study conducted in Andhra Pradesh, wherein, the mean arsenic content in pulses and vegetables was found as 0.013 and 0.269 mg/kg, respectively (Khandare et al., 2022). Similarly, Román‐Ochoa et al. (2021) reported that the mean arsenic content in beans was 0.02 mg/kg in Peru. Some studies correlated cooking with heavy metal content. For instance, Azi et al. (2018) revealed that cooking of vegetables significantly reduced the arsenic levels of vegetables compared to raw vegetables. However, the arsenic content in vegetables decreased with cooking in distilled water but increased with arsenic contaminated water (Rahman et al., 2011).

**TABLE 3:** Heavy metal content (mg/kg) in cooked beans and cooked vegetable preparations collected from selected urban and rural households of Ludhiana and Bathinda districts.

Heavy metals	Food group	Ludhiana		Bathinda		Permissible limits
		Urban	Rural	Urban	Rural	
Arsenic	Cooked beans	1.44 × 10^−5^ ± 8.00 × 10^−6a^	1.67 × 10^−6^ ± 3.70 × 10^−6b^	9.13 × 10^−6^ ± 1.79 × 10^−5ac^	3.68 × 10^−6^ ± 7.74 × 10^−6bc^	1.1 ppm^+^
	Cooked vegetable preparations	1.44 × 10^−5^ ± 1.56 × 10^−5ab^	1.53 × 10^−5^ ± 1.18 × 10^−5a^	5.73 × 10^−6^ ± 1.01 × 10^−5bc^	3.95 × 10^−6^ ± 1.21 × 10^−5c^	1.1 ppm^+^
Cadmium	Cooked beans	3.53 × 10^−5^ ± 4.35 × 10^−5a^	6.00 × 10^−7^ ± 2.32 × 10^−6b^	ND	ND	0.1 mg/kg^++^ 0.1 ppm^+^
	Cooked vegetable preparations	8.21 × 10^−5^ ± 5.60 × 10^−5a^	5.58 × 10^−5^ ± 3.87 × 10^−5a^	1.33 × 10^−5^ ± 2.14 × 10^−5b^	1.39 × 10^−5^ ± 3.07 × 10^−5b^	0.05–0.2 mg/kg^++^ 0.05–0.2 ppm^+^
Lead	Cooked beans	4.04 × 10^−4^ ± 5.43 × 10^−4^	4.38 × 10^−4^ ± 1.70 × 10^−3^	5.43 × 10^−4^ ± 1.79 × 10^−3^	ND	0.1 mg/kg^++^ 0.2 ppm^+^
	Cooked vegetable preparations	1.30 × 10^−3^ ± 4.40 × 10^−3^	2.98 × 10^−4^ ± 5.58 × 10^−4^	7.27 × 10^−5^ ± 1.06 × 10^−4^	ND	0.05–0.3 mg/kg^++^ 0.1–0.3 ppm^+^
Mercury	Cooked beans	9.09 × 10^−8^ ± 4.26 × 10^−7a^	ND	1.17 × 10^−4^ ± 1.91 × 10^−4b^	ND	
	Cooked vegetable preparations	2.61 × 10^−7^ ± 1.25 × 10^−6a^	ND	3.59 × 10^−5^ ± 7.81 × 10^−5b^	ND	

*Note*: Values are expressed as mean ± SD. “^a–c^” means within each column with different superscripts are significantly (*p* < .05) different. “^+^”: FSSAI (2011); “^++^”: Codex Alimentarius Commission (2019).

The mean cadmium concentration in cooked beans collected from selected urban (3.53 × 10^−5^ mg/kg) and rural (6.00 × 10^−7^ mg/kg) households of Ludhiana district differed significantly (*p* ≤ .0001). Cadmium was not detected from cooked beans of both urban and rural households of Bathinda district. The cadmium content in cooked beans from Ludhiana district was less than the permissible limit of 0.1 mg/kg (Codex Alimentarius Commission, 2019; FSSAI, 2011). Román‐Ochoa et al. (2021) reported that beans had 0.01 mg/kg of mean lead content in Peru. Similarly, Khandare et al. (2022) reported that the mean cadmium content in the samples of pulses from Andhra Pradesh was 0.013 mg/kg. The mean cadmium content in vegetable preparations from urban and rural households of Ludhiana district was 8.21 × 10^−5^ and 5.58 × 10^−5^ mg/kg, respectively, whereas, for Bathinda district, the respective values were 1.33 × 10^−5^ and 1.39 × 10^−5^ mg/kg. The cadmium content in vegetable preparations from both the districts differed significantly (*p* ≤ .0001). However, the cadmium content in vegetable preparations was less than the permissible limit of 0.005–0.2 mg/kg (Codex Alimentarius Commission, 2019; FSSAI, 2011) in both districts. Khandare et al. (2022) reported that the mean cadmium content in vegetables was 0.371 mg/kg. In another study, Ametepey et al. (2018) observed that cadmium content in different vegetable samples ranged from 0.01–0.07 mg/kg in Ghana. The cadmium content in cooked vegetable samples of present study was less than the values reported by Joshua et al. (2012). Kananke et al. (2015) showed that cooked vegetables had no significant reduction in cadmium content when compared to raw vegetables. It indicated that cooking had minimal effect on cadmium content of vegetables. In contrast, Azi et al. (2018) reported that cooking significantly reduced the cadmium content in vegetables.

In cooked beans, the mean lead content was 4.04 × 10^−4^ mg/kg for urban and 4.38 × 10^−4^ mg/kg for rural households of Ludhiana district. In Bathinda district, lead content in cooked beans from urban and rural households was 5.43 × 10^−4^ and 0.00 mg/kg, respectively. It was found that lead content in the samples of cooked beans from both the districts was within the permissible limit of 0.1 and 0.2 mg/kg as suggested by Codex Alimentarius Commission (2019) and FSSAI (2011), respectively. The mean lead content in vegetable preparations was 1.30 × 10^−3^ mg/kg for urban and 2.98 × 10^−4^ mg/kg for rural households of Ludhiana district, whereas, the value was 7.27 × 10^−5^ mg/kg for urban households of Bathinda district. The lead content in vegetable samples in both districts was less than the permissible limit of 0.05–0.3 mg/kg (Codex Alimentarius Commission, 2019) and 0.1–0.3 mg/kg (FSSAI, 2011). The lead content in the cooked samples in present study from both the districts was less than the lead in pulses and vegetables samples from India, Peru, and Iran (Khandare et al., 2022; Román‐Ochoa et al., 2021; Salehipour et al., 2015).

Average mercury content in cooked beans was 9.09 × 10^−8^ and 1.17 × 10^−4^ mg/kg for urban areas of Ludhiana and Bathinda district, respectively. The average mercury content in cooked vegetable preparations from urban households was 2.61 × 10^−7^ and 3.59 × 10^−5^ mg/kg for Ludhiana and Bathinda district, respectively. However, the mercury content in cooked beans and cooked vegetable preparations was not detected in rural households of both districts. The mean mercury content in present study was less than in pulses (3.4 × 10^−3^ mg/kg) of Peru (Román‐Ochoa et al., 2021). Similarly, Khandare et al. (2022) reported higher values of mercury content in pulse and vegetable samples in Andhra Pradesh, such as 0.006 and 0.019 mg/kg than the values reported in present study. The low mercury levels in cooked vegetable preparations indicated that cooking reduced the heavy metal content in vegetables. These results were supported by Azi et al. (2018), which revealed that cooking significantly reduced the mercury content in vegetables compared to raw ones.

### 3.2 Chronic daily intake of heavy metals from cooked beans and cooked vegetables

The CDI of heavy metals from cooked beans and cooked vegetable preparations among the selected urban and rural population of both districts is presented in Table 4.

**TABLE 4:** Chronic Daily Intake (CDI) of heavy metals (mg/kg/day) from cooked beans and cooked vegetable preparations by the selected urban and rural population of Ludhiana and Bathinda districts.

Heavy metals		Cooked beans				Cooked vegetable preparations			
		Ludhiana		Bathinda		Ludhiana		Bathinda	
		Urban	Rural	Urban	Rural	Urban	Rural	Urban	Rural
Arsenic									
Adult	Males	7.74 × 10^−9a^	4.84 × 10^−10b^	5.31 × 10^−9a^	2.13 × 10^−9b^	5.62 × 10^−8a^	3.85 × 10^−8ab^	1.32 × 10^−8c^	3.22 × 10^−8bc^
	Females	7.93 × 10^−9a^	7.12 × 10^−10b^	4.82 × 10^−9c^	2.27 × 10^−9bc^	6.71 × 10^−8a^	5.37 × 10^−8ab^	2.19 × 10^−8c^	3.04 × 10^−8bc^
Elderly	Males	8.31 × 10^−9a^	8.11 × 10^−10b^	5.71 × 10^−9ab^	2.07 × 10^−9ab^	6.67 × 10^−8^	6.19 × 10^−8^	2.40 × 10^−8^	1.77 × 10^−8^
	Females	9.94 × 10^−9^	–	7.04 × 10^−9^	–	4.92 × 10^−8^	3.88 × 10^−8^	1.94 × 10^−8^	2.14 × 10^−8^
Cadmium									
Adult	Males	2.20 × 10^−8a^	8.45 × 10^−11b^	–	–	3.13 × 10^−7a^	1.71 × 10^−7b^	3.97 × 10^−8c^	5.39 × 10^−8c^
	Females	1.88 × 10^−8a^	3.12 × 10^−10b^	–	–	3.76 × 10^−7a^	1.87 × 10^−7b^	5.40 × 10^−8c^	4.55 × 10^−8c^
Elderly	Males	2.67 × 10^−8a^	9.12 × 10^−10b^	–	–	4.20 × 10^−7a^	9.62 × 10^−8b^	5.18 × 10^−8b^	7.77 × 10^−9b^
	Females	3.30 × 10^−8^	–	–	–	3.78 × 10^−7a^	1.62 × 10^−7b^	6.00 × 10^−8b^	4.29 × 10^−8b^
Lead									
Adult	Males	2.43 × 10^−7^	9.06 × 10^−8^	3.35 × 10^−7^	–	7.80 × 10^−6a^	7.13 × 10^−7b^	1.92 × 10^−7b^	–
	Females	2.18 × 10^−7^	1.45 × 10^−7^	3.36 × 10^−7^	–	6.35 × 10^−6a^	8.95 × 10^−7b^	3.20 × 10^−7b^	–
Elderly	Males	2.99 × 10^−7^	–	3.32 × 10^−7^	–	6.68 × 10^−6^	3.79 × 10^−7^	3.04 × 10^−7^	–
	Females	4.56 × 10^−7^	–	7.04 × 10^−7^	–	2.26 × 10^−6a^	5.18 × 10^−7b^	1.39 × 10^−7b^	–
Mercury									
Adult	Males	7.00 × 10^−11a^	–	5.48 × 10^−8b^	–	7.88 × 10^−10a^	–	5.39 × 10^−8b^	–
	Females	7.07 × 10^−11a^	–	5.77 × 10^−8b^	–	6.90 × 10^−10a^	–	1.15 × 10^−7b^	–
Elderly	Males	7.62 × 10^−11a^	–	1.00 × 10^−7b^	–	–	–	1.20 × 10^−7^	–
	Females	2.06 × 10^−10a^	–	1.01 × 10^−7b^	–	–	–	1.14 × 10^−7^	–

*Note*: Values are expressed as mean intakes of selected heavy metals. “^a–c^” means within each column with different superscripts are significantly (*p* < .05) different.

#### 3.2.1 Cooked beans

The mean CDI of arsenic from cooked beans was significantly (*p* ≤ .001) higher in urban adult males of Ludhiana (7.74 × 10^−9^ mg/kg/day) and Bathinda (5.31 × 10^−9^ mg/kg/day) districts compared to their rural counterparts. The mean CDI of arsenic from cooked beans in urban adult females of Ludhiana district was significantly (*p* ≤ .001) higher (7.93 × 10^−9^ mg/kg/day). A significant (*p* = .047) difference in arsenic intake was observed between urban (8.31 × 10^−9^ mg/kg/day) and rural (8.11 × 10^−10^ mg/kg/day) elderly males of Ludhiana district. In Ludhiana district, a significantly (*p* ≤ .001) higher mean CDI of cadmium from cooked beans was observed in urban adult males (2.20 × 10^−8^ mg/kg/day) than rural adult males (8.45 × 10^−11^ mg/kg/day). However, the CDI of cadmium from cooked beans was not estimated for different groups of Bathinda district as cadmium was not detected in samples of cooked beans from selected urban and rural households of Bathinda district. The mean CDI of cadmium from cooked beans was significantly (*p* ≤ .001) higher in urban adult females (1.88 × 10^−8^ mg/kg/day) in Ludhiana district compared to their rural counterparts. A significant difference (*p* = .002) was observed between urban (2.67 × 10^−8^ mg/kg/day) and rural (9.12 × 10^−10^ mg/kg/day) elderly males of Ludhiana district regarding the mean CDI of cadmium. The mean CDI of cadmium was 3.30 × 10^−8^ mg/kg/day in urban elderly females of Ludhiana district. The mean CDI of lead from cooked beans was 2.43 × 10^−7^ versus 9.06 × 10^−8^ and 2.18 × 10^−7^ versus 1.45 × 10^−7^ mg/kg/day for urban versus rural adult males and females of Ludhiana district, respectively. In Bathinda district, the mean CDI was 3.35 × 10^−7^ and 3.36 × 10^−7^ mg/kg/day for urban adult males and females, respectively. The mean CDI of lead among elderly males and females of Ludhiana versus Bathinda district was 2.99 × 10^−7^ and 4.56 × 10^−7^ versus 3.32 × 10^−7^ and 7.04 × 10^−7^ mg/kg/day, respectively. The mean CDI of mercury from cooked beans was significantly (*p* ≤ .001) higher in urban adult males (5.48 × 10^−8^ mg/kg/day) and females (5.77 × 10^−8^ mg/kg/day) in Bathinda district than in their urban counterparts in Ludhiana district. A significant (*p* = .009) difference was found between the mean CDI of urban elderly males of Ludhiana (7.62 × 10^−11^ mg/kg/day) and Bathinda (1.00 × 10^−7^ mg/kg/day) districts. A significantly (*p* = .001) higher mean CDI of mercury was found in urban elderly females of Bathinda district (1.01 × 10^−7^ mg/kg/day). Cadmium was not detected in the samples of cooked beans collected from urban and rural areas of Bathinda district. Similarly, lead was not detected in cooked beans of rural areas of Bathinda district while mercury was not detected in cooked beans of rural households in districts indicating that rural population is safer in terms of heavy metals namely, cadmium, lead, and mercury in comparison to their urban counterparts. Román‐Ochoa et al. (2021) revealed that from pulses, CDI of arsenic, lead, mercury, and cadmium was 7.3 × 10^−7^, 3.7 × 10^−7^, 1.2 × 10^−7^, and 3.7 × 10^−7^ mg/kg/day, respectively, in Peru. In another study, Khandare et al. (2022) reported that average daily intake of lead from pulses was 0.1 × 10^−3^ mg/kg among adults of Andhra Pradesh. The CDI of arsenic, cadmium, and lead from cooked beans in both the districts was lower than respective values of pulses from Bangladesh (Islam et al., 2014).

#### 3.2.2 Cooked vegetable preparations

The common vegetables consumed during the survey period were spinach, mustard leaves, fenugreek leaves, cauliflower, cabbage, carrot, peas, pumpkin, bottle gourd, potato, onion, tomato, lady finger, apple gourd etc., which were most often consumed by the selected households of urban and rural populations of Ludhiana and Bathinda district. The CDI of heavy metals from these cooked vegetable preparations is presented in Table 4. The mean CDI of arsenic from cooked vegetable preparations showed a significant (*p* ≤ .001) difference between urban adult males of Ludhiana (5.62 × 10^−8^ mg/kg/day) and Bathinda (1.32 × 10^−8^ mg/kg/day) districts. Similarly, a significant (*p* ≤ .001) difference was observed between the mean CDI of arsenic in urban adult females of Ludhiana (6.71 × 10^−8^ mg/kg/day) and Bathinda (2.19 × 10^−8^ mg/kg/day) districts. The mean CDI of cadmium from cooked vegetable preparations was significantly (*p* ≤ .001) higher in urban adult males of Ludhiana district (3.13 × 10^−7^ mg/kg/day). The mean CDI of cadmium in urban adult females of Ludhiana district (3.76 × 10^−7^ mg/kg/day) was significantly (*p* ≤ .001) higher than their rural counterparts and both urban and rural adult females of Bathinda district. A significantly (*p* ≤ .001) higher mean CDI of cadmium was found in urban elderly males (4.20 × 10^−7^ mg/kg/day) and females (3.78 × 10^−7^ mg/kg/day) of Ludhiana district. A significantly (*p* = .005) higher mean CDI of lead was observed in adult males of Ludhiana district (7.80 × 10^−6^ mg/kg/day). The mean CDI of lead was significantly (*p* = .022) higher in urban adult females of Ludhiana district (6.35 × 10^−6^ mg/kg/day). The urban elderly females of Ludhiana district had significantly (*p* ≤ .001) higher mean CDI of lead (2.26 × 10^−6^ mg/kg/day) than others. Khandare et al. (2022) conducted a study in Andhra Pradesh, which showed that average daily intake of arsenic, lead, and cadmium from vegetables was 0.1 × 10^−3^, 0.2 × 10^−3^, and 0.1 × 10^−3^ mg/kg among adults, respectively; however, these values were higher than the present study. Similarly, Salehipour et al. (2015) also reported slightly higher CDI values of lead from vegetables among adults and elderly population of Iran compared to present study. According to Fu and Cui (2013), the bioaccessibility and bioavailability of cadmium and lead were higher for raw vegetables compared to cooked vegetables, thus intake of both heavy metals higher from raw vegetables but less from cooked vegetables. Thus, consuming cooked vegetables decreased heavy metal intake and associated health risks. The mean CDI of mercury from cooked vegetable preparations was significantly (*p* = .021) higher in urban adult males of Bathinda district (5.39 × 10^−8^ mg/kg/day) than in their urban counterparts in Ludhiana district. Similarly, urban adult females in Bathinda district had significantly (*p* = .015) higher mean CDI of mercury (1.15 × 10^−7^ mg/kg/day). The mean CDI of mercury in urban elderly males and females of Bathinda district was 1.20 × 10^−7^ and 1.14 × 10^−7^ mg/kg/day, respectively, however, their rural counterparts were safer as no mercury was detected in their cooked vegetable samples.

### 3.3 Non‐carcinogenic risk

The THQ and HI of heavy metals through the consumption of cooked beans and cooked vegetable preparations among the selected urban and rural populations of both districts have been presented in Table 5.

**TABLE 5:** Target Hazard Quotient (THQ) and Hazard Index (HI) of heavy metals from cooked beans and cooked vegetable preparations by the selected urban and rural population of Ludhiana and Bathinda districts.

Heavy metals		Cooked beans				Cooked vegetable preparations			
		Ludhiana		Bathinda		Ludhiana		Bathinda	
		Urban	Rural	Urban	Rural	Urban	Rural	Urban	Rural
Target hazard quotient									
*Arsenic*									
Adult	Males	2.58 × 10^−5a^	1.61 × 10^−6b^	1.77 × 10^−5a^	7.10 × 10^−6b^	1.87 × 10^−4a^	1.28 × 10^−4ab^	4.41 × 10^−5c^	1.07 × 10^−4bc^
	Females	2.64 × 10^−5a^	2.37 × 10^−6b^	1.61 × 10^−5c^	7.56 × 10^−6bc^	2.24 × 10^−4a^	1.79 × 10^−4ab^	7.30 × 10^−5c^	1.01 × 10^−4bc^
Elderly	Males	2.77 × 10^−5a^	2.70 × 10^−6b^	1.90 × 10^−5ab^	6.89 × 10^−6ab^	2.22 × 10^−4^	2.06 × 10^−4^	7.99 × 10^−5^	5.90 × 10^−5^
	Females	3.31 × 10^−5^	–	2.35 × 10^−5^	–	1.64 × 10^−4^	1.29 × 10^−4^	6.48 × 10^−5^	7.15 × 10^−5^
*Cadmium*									
Adult	Males	2.20 × 10^−4a^	8.45 × 10^−7b^	–	–	3.13 × 10^−3a^	1.71 × 10^−3b^	3.97 × 10^−4c^	5.39 × 10^−4c^
	Females	1.88 × 10^−4a^	3.12 × 10^−6b^	–	–	3.76 × 10^−3a^	1.87 × 10^−3b^	5.40 × 10^−4c^	4.55 × 10^−4c^
Elderly	Males	2.67 × 10^−4a^	9.12 × 10^−6b^	–	–	4.20 × 10^−3a^	9.62 × 10^−4b^	5.18 × 10^−4b^	7.77 × 10^−5b^
	Females	3.30 × 10^−4^	–	–	–	3.78 × 10^−3a^	1.62 × 10^−3b^	6.00 × 10^−4b^	4.29 × 10^−4b^
*Lead*									
Adult	Males	6.95 × 10^−5^	2.59 × 10^−5^	9.57 × 10^−5^	–	2.23 × 10^−3a^	2.04 × 10^−4b^	5.50 × 10^−5b^	–
	Females	6.22 × 10^−5^	4.13 × 10^−5^	9.61 × 10^−5^	–	1.81 × 10^−3a^	2.56 × 10^−4b^	9.15 × 10^−5b^	–
Elderly	Males	8.55 × 10^−5^	–	9.49 × 10^−5^	–	1.91 × 10^−3^	1.08 × 10^−4^	8.68 × 10^−5^	–
	Females	1.30 × 10^−4^	–	2.01 × 10^−4^	–	6.47 × 10^−4a^	1.48 × 10^−4b^	3.98 × 10^−5b^	–
Hazard index									
Adult	Males	3.15 × 10^−4a^	2.83 × 10^−5b^	1.13 × 10^−4a^	7.10 × 10^−6b^	5.55 × 10^−3a^	2.05 × 10^−3b^	4.96 × 10^−4b^	6.47 × 10^−4b^
	Females	2.76 × 10^−4a^	4.68 × 10^−5ab^	1.12 × 10^−4a^	7.56 × 10^−6b^	5.80 × 10^−3a^	2.31 × 10^−3b^	7.05 × 10^−4b^	5.56 × 10^−4b^
Elderly	Males	3.80 × 10^−4^	1.18 × 10^−5^	1.14 × 10^−4^	6.89 × 10^−6^	6.33 × 10^−3^	1.28 × 10^−3^	6.85 × 10^−4^	1.37 × 10^−4^
	Females	4.94 × 10^−4^	–	2.24 × 10^−4^	–	4.59 × 10^−3a^	1.90 × 10^−3b^	7.04 × 10^−4b^	5.01 × 10^−4b^

*Note*: Values are expressed as mean. “^a–c^” means within each column with different superscripts are significantly (*p* < .05) different.

#### 3.3.1 Cooked beans

In Ludhiana district, the mean THQ of arsenic from cooked beans among urban (2.58 × 10^−5^) and rural (1.61 × 10^−6^) adult males was significantly (*p* ≤ .001) different from each other. Similarly, in Bathinda district, the corresponding mean THQ values of 1.77 × 10^−5^ and 7.10 × 10^−6^ showed a significant (*p* ≤ .001) difference. Ingestion rate, frequency of intake, length of food consumption, and weight of people consuming food are some variables that affect risk assessment (Sharafi et al., 2019). A significantly (*p* ≤ .001) higher THQ of arsenic (2.64 × 10^−5^) was reported in urban adult females of Ludhiana district. In Ludhiana district, the mean THQ of arsenic in urban (2.77 × 10^−5^) and rural (2.70 × 10^−6^) elderly males was significantly (*p* = .047) different. The mean THQ of arsenic among elderly females did not differ significantly. In Ludhiana district, the mean THQ of cadmium from cooked beans was significantly (*p* ≤ .001) different between urban (2.20 × 10^−4^) and rural (8.45 × 10^−7^) adult males. As cadmium was not detected in both urban and rural cooked samples of cooked beans of Bathinda district, THQ was not calculated. The urban and rural adult females of Ludhiana district had significantly (*p* ≤ .001) different mean THQ of cadmium, that is, 1.88 × 10^−4^ and 3.12 × 10^−6^, respectively. The THQ of cadmium was 2.67 × 10^−4^ and 9.12 × 10^−6^ for urban and rural elderly males of Ludhiana district, which were significantly (*p* = .002) different from each other. The THQ of cadmium for rural elderly females of Ludhiana district were not derived due to non‐detectable cadmium content in cooked beans of the group. The mean THQ of cadmium for urban elderly females of Ludhiana district was 3.30 × 10^−4^. The mean THQ of lead from cooked beans was not estimated for the selected rural population of Bathinda district and rural elderly males of Ludhiana district due to the absence of lead in their samples of cooked beans. A non‐significant difference was observed for both age groups.

The average Hazard Index of heavy metals from cooked beans, that is, 3.15 × 10^−4^ versus 2.83 × 10^−5^ and 1.13 × 10^−4^ versus 7.10 × 10^−6^ in urban versus rural adult males of Ludhiana and Bathinda district was significantly (*p* = .002) different. In Bathinda district, the mean HI in urban adult females (1.12 × 10^−4^) was significantly (*p* = .035) higher than their rural counterparts. For rest of the population groups, no significant difference was observed. The mean HI for the selected population of both Ludhiana and Bathinda districts indicated that the population was safe from non‐carcinogenic risk from heavy metal consumption through cooked beans. Khandare et al. (2022) reported that THQ of cadmium (0.006 and 0.012), lead (0.136 and 0.086), arsenic (0.018 and 0.026), and HI (0.424 and 0.456) among adults and children, respectively, were less than 1, thereby indicating that pulses were not responsible for non‐carcinogenic risk.

#### 3.3.2 Cooked vegetable preparations

The average THQ of arsenic from cooked vegetable preparations was significantly (*p* ≤ .001) higher in urban adult males (1.87 × 10^−4^) and females (2.24 × 10^−4^) of Ludhiana district compared to their urban counterparts in Bathinda district. No significant difference in THQ of arsenic was found among the selected elderly populations of urban and rural areas of the two districts. The mean THQ of cadmium was significantly (*p* ≤ .001) higher, that is, 3.13 × 10^−3^ and 3.76 × 10^−3^ among urban adult males and females of Ludhiana district, respectively. Humans can readily consume these dangerous pollutants, which are frequently present on the surface of fresh vegetables and in their tissues. Numerous studies found that eating vegetables rich in Pb, Cu, and Cd concentrations increased the risk of gastrointestinal cancer. Vegetables produced on contaminated soil all over the world accumulate high amounts of heavy metals (Bigdeli & Seilsepour, 2008; Chang et al., 2014; Gupta et al., 2021; Ismail et al., 2014; Türkdoğan et al., 2003). Similarly, the urban elderly males and females of Ludhiana district also showed significantly (*p* ≤ .001) higher average THQ of cadmium, that is, 4.20 × 10^−3^ and 3.78 × 10^−3^, respectively. In urban adult males of Ludhiana district, a significantly (*p* = .005) higher mean THQ of lead (2.23 × 10^−3^) was observed. Similarly, the urban adult females of Ludhiana district also had significantly (*p* = .022) higher mean THQ of lead (1.81 × 10^−3^) as compared to other groups. A significantly (*p* ≤ .001) higher average THQ was observed for urban elderly females of Ludhiana district (6.47 × 10^−4^). No significant difference was found among elderly males of both districts. When present in food in concentrations over their permissible levels, heavy metals like chromium, copper, manganese, and zinc are said to be able to produce non‐carcinogenic dangers, such as neurologic problems and liver illness (Ekhator et al., 2017).

The mean Hazard Index from cooked vegetable preparations was found to be significantly (*p* ≤ .001) higher in urban adult males (5.55 × 10^−3^) and females (5.80 × 10^−3^) of Ludhiana district. A significant (*p* ≤ .001) difference was observed between urban elderly females of Ludhiana district (4.59 × 10^−3^) and other groups. However, in both the districts, the selected population was found to be safe from non‐carcinogenic risk due to combined effect of arsenic, lead, cadmium, and mercury. Khandare et al. (2022) reported that although HQ of arsenic, lead, and cadmium among adults were less than 1 but the combined effect of heavy metals present in vegetables becomes a reason for non‐carcinogenic risk among adults from Andhra Pradesh with HI of 1.003. Ametepey et al. (2018) reported that HQ of cadmium from vegetables among adults ranged between 2.51 and 3.14 in Ghana, which was a significant concern for the population. Along with single metal, the combined effect of heavy metals also showed a serious impact on population of Ghana, Vietnam, Algeria, Nigeria, and Iran as HI was more than 1 (Ametepey et al., 2018; Bui et al., 2016; Cherfi et al., 2014; Kharazi et al., 2021; Patrick‐Iwuanyanwu & Chioma, 2017).

### 3.4 Carcinogenic risk

The CR and TCRI of heavy metals from cooked beans and cooked vegetable preparations in the selected population of Ludhiana and Bathinda district have been presented in Table 6.

**TABLE 6:** Carcinogenic risk (CR) and Total Carcinogenic Risk Index (TCRI) of heavy metals from cooked beans and cooked vegetable preparations by the selected urban and rural population of Ludhiana and Bathinda districts.

Heavy metals		Cooked beans				Cooked vegetable preparations			
		Ludhiana		Bathinda		Ludhiana		Bathinda	
		Urban	Rural	Urban	Rural	Urban	Rural	Urban	Rural
Carcinogenic risk (CR)									
*Arsenic*									
Adult	Males	1.16 × 10^−8a^	7.26 × 10^−10b^	7.96 × 10^−9ac^	3.20 × 10^−9bc^	8.43 × 10^−8a^	5.78 × 10^−8ab^	1.98 × 10^−8c^	4.83 × 10^−8bc^
	Females	1.19 × 10^−8a^	1.07 × 10^−9b^	7.23 × 10^−9c^	3.40 × 10^−9bc^	1.01 × 10^−7a^	8.05 × 10^−8ab^	3.28 × 10^−8c^	4.56 × 10^−8bc^
Elderly	Males	1.25 × 10^−8a^	1.22 × 10^−9b^	8.56 × 10^−9ab^	3.10 × 10^−9ab^	1.00 × 10^−7^	9.29 × 10^−8^	3.60 × 10^−8^	2.65 × 10^−8^
	Females	1.49 × 10^−8^	–	1.06 × 10^−8^	–	7.39 × 10^−8^	5.82 × 10^−8^	2.92 × 10^−8^	3.22 × 10^−8^
*Cadmium*									
Adult	Males	1.34 × 10^−7a^	5.15 × 10^−10b^	–	–	1.91 × 10^−6a^	1.05 × 10^−6b^	2.42 × 10^−7c^	3.29 × 10^−7c^
	Females	1.14 × 10^−7a^	1.90 × 10^−9b^	–	–	2.30 × 10^−6a^	1.14 × 10^−6b^	3.30 × 10^−7c^	2.78 × 10^−7c^
Elderly	Males	1.63 × 10^−7a^	5.56 × 10^−9b^	–	–	2.56 × 10^−6a^	5.87 × 10^−7b^	3.16 × 10^−7b^	4.74 × 10^−8b^
	Females	2.02 × 10^−7^	–	–	–	2.31 × 10^−6a^	9.89 × 10^−7b^	3.66 × 10^−7b^	2.62 × 10^−7b^
*Lead*									
Adult	Males	2.07 × 10^−9^	7.70 × 10^−10^	2.85 × 10^−9^	–	6.63 × 10^−8a^	6.06 × 10^−9b^	1.64 × 10^−9b^	–
	Females	1.85 × 10^−9^	1.23 × 10^−9^	2.86 × 10^−9^	–	5.40 × 10^−8a^	7.61 × 10^−9b^	2.72 × 10^−9b^	–
Elderly	Males	2.54 × 10^−9^	–	2.82 × 10^−9^	–	5.68 × 10^−8^	3.22 × 10^−9^	2.58 × 10^−9^	–
	Females	3.88 × 10^−9^	–	5.98 × 10^−9^	–	1.92 × 10^−8a^	4.41 × 10^−9b^	1.18 × 10^−9b^	–
Total Carcinogenic Risk Index (TCRI)									
Adult	Males	1.48 × 10^−7a^	2.01 × 10^−9b^	1.08 × 10^−8b^	3.20 × 10^−9b^	2.06 × 10^−6a^	1.11 × 10^−6b^	2.64 × 10^−7c^	3.77 × 10^−7c^
	Females	1.28 × 10^−7a^	4.20 × 10^−9b^	1.01 × 10^−8b^	3.40 × 10^−9b^	2.45 × 10^−6a^	1.23 × 10^−6b^	3.65 × 10^−7c^	3.23 × 10^−7c^
Elderly	Males	1.78 × 10^−7^	6.78 × 10^−9^	1.14 × 10^−8^	3.10 × 10^−9^	2.72 × 10^−6a^	6.83 × 10^−7b^	3.55 × 10^−7b^	7.40 × 10^−8b^
	Females	2.20 × 10^−7a^	–	1.65 × 10^−8b^	–	2.40 × 10^−6a^	1.05 × 10^−6b^	3.96 × 10^−7b^	2.94 × 10^−7b^

*Note*: Values are expressed as Mean. “^a–c^” means within each column with different superscripts are significantly (*p* < .05) different.

#### 3.4.1 Cooked beans

The mean CR of arsenic from cooked beans was significantly (*p* ≤ .001) higher in urban adult males of Ludhiana district (1.16 × 10^−8^) as compared to rural adult males of both Ludhiana and Bathinda districts. For urban adult females of Ludhiana district, a significantly (*p* ≤ .001) higher mean CR (1.19 × 10^−8^) was observed.

In Ludhiana district, a significant (*p* = .047) difference was observed between the mean CR of arsenic in urban (1.25 × 10^−8^) and rural (1.22 × 10^−9^) elderly males. However, the carcinogenic risk from arsenic did not exceed its permissible limit in the selected urban and rural populations of both districts. The urban adult males (1.34 × 10^−7^) and females (1.14 × 10^−7^) of Ludhiana district had significantly (*p* ≤ .001) higher mean CR of cadmium from cooked beans than their rural counterparts. The average CR of cadmium in urban elderly males of Ludhiana district (1.63 × 10^−7^) was significantly (*p* = .002) higher. Only 8% urban elderly females of Ludhiana district were at risk. The results showed that the selected population was not at carcinogenic risk due to cadmium from cooked beans. The average CR of lead from cooked beans was not significantly different between groups for the selected population of both districts. The mean CR values indicated that the population was safe from carcinogenic risk associated with cooked beans as they were less than 10^−6^. However, in Bangladesh, pulses consumption leads to carcinogenic risk related to arsenic and lead among adults as CR of lead and arsenic exceed the threshold levels (Islam et al., 2014; Kormoker et al., 2020).

The mean TCRI from cooked beans showed a significantly higher values in adult males (1.48 × 10^−7^) and females (1.28 × 10^−7^) of Ludhiana district. A significant (*p* = .042) difference in the average TCRI of urban elderly females of Ludhiana (2.20 × 10^−7^) and Bathinda (1.65 × 10^−8^) districts was found. The results indicated that even after the combined effect of heavy metals from cooked beans, the selected populations of both districts were safe from carcinogenic risk.

#### 3.4.2 Cooked vegetable preparations

The average CR of arsenic in urban adult males (8.43 × 10^−8^) and females (1.01 × 10^−7^) of Ludhiana district were significantly (*p* ≤ .001) higher than their urban and rural counterparts from Bathinda district. The other population groups did not significantly differ within or between the districts. However, the results revealed that the selected populations of both districts were safe from carcinogenic risk. The mean CR of cadmium from cooked vegetable preparations was significantly (*p* ≤ .001) higher in both urban versus rural adult males (1.91 × 10^−6^ vs. 1.05 × 10^−6^) and females (2.30 × 10^−6^ vs. 1.14 × 10^−6^) of Ludhiana district compared to their counterparts from Bathinda district. In Ludhiana district, 69% and 74% urban adult male and females along with 45 and 42% rural adult male and females were at risk, respectively. While in Bathinda district, among adults, 7% and 9% urban and 14% and 13% rural males and females had cancer risk, respectively. A significantly (*p* ≤ .001) higher mean CR of cadmium was found in urban elderly males (2.56 × 10^−6^) and females (2.31 × 10^−6^) of Ludhiana district. It was found that both urban and rural adult males and females and only urban elderly males and females of Ludhiana district had carcinogenic risk from cadmium present in cooked vegetable samples. Metals like As, Cd, and Pb, as well as other necessary and non‐essential metals, are typically found in soil. Several research studies have been published about the metal contamination of agricultural soils. Additionally, anthropogenic activity, like the use of chemical fertilizer, may have increased the metal concentrations in soil above natural levels, in addition to the geological characteristics of soils. When cultivated in such soils, vegetables have the ability to accumulate numerous chemicals and contaminants in various sections of the plant through foliar or root uptake (Babandi et al., 2020; Baghaie & Fereydoni, 2019). In Ludhiana district, 75% (males) and 85% (females) urban elderly subjects and 33% rural elderly females had cancer risk. However, in Bathinda district only 6% (urban) and 10% (rural) elderly females were at risk.

The average CR value of lead from cooked vegetable preparations was significantly (*p* = .005) higher in urban adult males of Ludhiana district (6.63 × 10^−8^). Only 1% urban adult females of Ludhiana district were at risk. In Ludhiana district, urban adult females also had significantly (*p* = .022) higher mean CR of lead (5.40 × 10^−8^). A significantly (*p* ≤ .001) higher mean CR of lead was found in urban elderly females of Ludhiana district (1.92 × 10^−8^). These results indicated that the selected population in both the districts was not at carcinogenic risk. In Bangladesh, CR of arsenic and lead present in vegetables exceeded the standard limit indicating higher risk of cancer among adults (Islam et al., 2016; Kormoker et al., 2020). Similar results were found in Andhra Pradesh, India, which showed a carcinogenic risk among adults and children from arsenic, lead, and cadmium due to presence of heavy metals in the vegetables (Khandare et al., 2022).

The average TCRI from cooked vegetable preparations in both urban versus rural adult males (2.06 × 10^−6^ vs. 1.11 × 10^−6^) and females (2.45 × 10^−6^ vs. 1.23 × 10^−6^) of Ludhiana district were significantly (*p* ≤ .001) higher than the corresponding values in Bathinda district. In Ludhiana district, urban elderly males and females had significantly higher mean TCRI of 2.72 × 10^−6^ and 2.40 × 10^−6^, respectively. In both districts, the mean TCRI in the selected population was less than its permissible limit (10^−4^).

## 4 CONCLUSIONS

The present study concluded that arsenic, lead, cadmium, and mercury content in the samples of cooked beans and cooked vegetable preparations collected from selected urban and rural households of Ludhiana and Bathinda districts were within permissible limit, indicating that they were safe. It was also observed that the male and female adults and elderly population of both districts were safe from non‐carcinogenic risk associated with arsenic, lead, and cadmium present in cooked beans and cooked vegetable preparations. However, the urban adult and elderly population and rural adult population of Ludhiana district had cancer risk due to cadmium present in cooked vegetable samples while other groups were safe. Consumption of cooked beans were also safe as no carcinogenic risk was observed from individual or combination of heavy metals present in samples. It revealed that though consumption of beans was safe for population of both districts, but preventive measures must be taken in order to reduce cadmium content in vegetable samples for better health.

## AUTHOR CONTRIBUTIONS

**Vineeta Kharkwal:** Conceptualization (equal); formal analysis (equal); investigation (equal); methodology (equal); writing – original draft (equal); writing – review and editing (equal). **Monika Choudhary:** Investigation (equal); supervision (equal); writing – review and editing (equal). **Kiran Bains:** Conceptualization (equal); investigation (equal); methodology (equal); supervision (equal); writing – review and editing (equal). **Mahendra Bishnoi:** Conceptualization (equal); investigation (equal); methodology (equal); supervision (equal).

## FUNDING INFORMATION

No funding was received for conducting this study.

## CONFLICT OF INTEREST STATEMENT

Authors have no conflict of interest.

## ETHICS STATEMENT

The questionnaire and methodology for this study was approved by the Ethical committee of the Punjab Agricultural University, Ludhiana, Punjab, India (Endst. No. DR.III.AU.2019/9589.98).

## CONSENT TO PARTICIPATE STATEMENT

Verbal informed consent was obtained from participants prior to interview.

## Data Availability

The data that support the findings of this study are available from the corresponding author upon reasonable request.
